# Advanced Prostate Cancer Presenting as Hemolytic Uremic Syndrome

**DOI:** 10.1155/2013/459618

**Published:** 2013-04-30

**Authors:** R. Ramos, F. Lopes, T. Rodrigues, N. Rolim, I. Rodrigues, H. Monteiro

**Affiliations:** ^1^Urology Department, Portuguese Institute of Oncology, 1099 Lisbon, Portugal; ^2^Urology Department, Egas Moniz Hospital, CHLO, 1349 Lisbon, Portugal; ^3^Nephrology Department, Santa Cruz Hospital, CHLO, 2790 Carnaxide, Portugal

## Abstract

*Introduction*. Hemolytic uremic syndrome (HUS) is characterized by endothelial dysfunction, consumption thrombocytopenia, microangiopathic hemolytic anemia, and acute renal failure. HUS generally has a dismal prognosis, except when associated with gastroenteritis caused by verotoxin-producing bacteria. Cancer associated HUS is uncommon, and there are only scarce reports on prostate cancer presenting with HUS. *Case Presentation*. A 72-year-old man presented to the emergency department with oliguria, hematuria, and hematemesis. Clinical evaluation revealed acute renal failure, hemolysis, normal blood-clotting studies, and prostate-specific antigen value of 1000 ng/mL. The patient was started on hemodialysis, ultrafiltration with plasma exchange, and androgen blockade with bicalutamide and completely recovered from HUS. The authors review the 14 published cases on this association. *Conclusion*. The association of HUS and prostate cancer occurs more frequently in patients with high-grade, clinically advanced prostate cancer. When readily recognized and appropriately treated, HUS does not seem to worsen prognosis in prostate cancer patients.

## 1. Introduction

Hemolytic uremic syndrome (HUS) is a potentially life-threatening disease belonging to the spectrum of thrombotic microangiopathies. It is characterized by endothelial dysfunction which leads to consumption thrombocytopenia, microangiopathic hemolytic anemia, and acute renal failure. Neurologic abnormalities and fever may occur in the most severe cases. HUS etiology is variable and frequently idiopathic. While cases caused by infection with verotoxin-producing Escherichia coli and numerous drugs (e.g. mitomycin; ticlopidine) are well-documented, the association with adenocarcinoma neoplasms and specifically prostate cancer is rare. 

We review the 14 cases reported in the literature and add a new one. This paper pretends to highlight the apparent clinical homogeneity of this association.

## 2. Case Presentation

A 72-year-old man presented to the emergency department with hematemesis, hematuria, and oliguria. He had no complaints of diarrhea and was not taking any medication. On observation, there was no fever, purpura, or hypertension. Laboratory investigation revealed mild hemolytic anemia (hemoglobin 11.8 g/dL, total bilirubin 2.23 mg/dL, direct bilirubin 0.35 mg/dL, lactate dehydrogenase 3000 U/L, and haptoglobin 20.0 mg/dL), thrombocytopenia (platelets 54 × 10^9^/L) and renal failure (serum creatinine 6.76 mg/dL, urea 190 mg/dL). The serum sodium was 139 mmol/L, and potassium was unmeasurable due to hemolysis. The blood-clotting studies were normal (prothrombin time 12.1 seconds, activated partial thromboplastin time 26.8 seconds, and fibrinogen 2.2 g/L). The blood smear revealed fragmented erthrocytes and schistocytes. His prostate-specific antigen (PSA) was 1000 ng/mL. On imaging studies, the chest radiograph and renal ultrasound were normal.

These findings excluded disseminated intravascular coagulation and obstructive renal failure. The digital rectal examination showed a large, hard, and fixed prostate with an irregular surface. HUS and prostate cancer were diagnosed, and the patient was admitted to the nephrology department. Serum creatinine peaked at 10.49 mg/dL. A total of eight sessions of hemodialysis and seven sessions of plasmapheresis were completed, and the patient was started on bicalutamide 50 mg/day. Daily urine output increased after the third session of hemodialysis.

The blood count and renal function progressively improved, and the patient was discharged from hospital after 15 days with a hemoglobin of 9.8 g/dL, platelet count of 412 × 10^9^/L, and a serum creatinine of 6.94 mg/dL.

A prostate biopsy confirmed a bilateral Gleason 9 (4 + 5) prostate cancer, and a bone scan revealed disseminated metastases. Goserelin depot was associated with daily bicalutamide, and at an 18-months follow-up the HUS has not recurred and the patient is clinically stable with a serum creatinine of 0.96 mg/dL and a PSA of 4.2 ng/mL.

## 3. Discussion

At the best of our knowledge, 14 cases of HUS associated with prostate cancer have been reported in the literature with an average age of 72.6 ± 8.0 years and a follow-up ranging from 3 months to 7 years [1–10].

Disease stage was advanced with 13 patients having metastatic disease. One patient died due to progression of prostate cancer [1] and another from HUS complications [2]. 

Reports published after PSA measurement became available generally reveal high PSA values ranging from 59 to >1000 ng/mL [2, 3]. 

The main presenting symptoms were secondary to renal impairment, namely, oliguria and vomiting. All patients were treated with hemodialysis alone or complemented with plasmapheresis with 11 out of 14 completely recovering renal function; three cases had incomplete or no response [2, 3].

The HUS recurrence rate in patients with prostate cancer seems low as only three cases had a second event of HUS [6, 7, 9].

Our case apparently reveals the typical characteristics of this association: high grade disseminated prostate cancer with a good response to conventional HUS therapy. HUS has been reported in association with mucin-producing adenocarcinoma of the breast, gastrointestinal tract, and prostate [11, 12]. One may argue that the coexistence of prostate cancer and HUS is merely a result of chance, but the apparent homogeneity and benign clinical course of the HUS may represent a still unrecognized physiopathologic link.

HUS when readily recognized and treated does not seem to determine a worse prognosis to prostate cancer patients.
